# Piloting the Integration of Non-Dispensing Pharmacists in the Australian General Practice Setting: A Process Evaluation

**DOI:** 10.5334/ijic.3293

**Published:** 2018-04-18

**Authors:** Helen Benson, Daniel Sabater-Hernández, Shalom I. Benrimoj, Kylie A. Williams

**Affiliations:** 1 Graduate School of Health, University of Technology Sydney, AU; 2 Academic Centre in Pharmaceutical Care, University of Granada, ES

**Keywords:** process evaluation, pharmacist, integration, general practice, primary care, collaborative care

## Abstract

**Introduction:**

This process evaluation examined the circumstances affecting implementation, intervention design and situational context of the twelve week pilot phase of a project integrating five pharmacists into twelve general practice sites in Western Sydney.

**Description of Care Practice:**

This study used a mixed method study design using qualitative data obtained from semi-structured interviews and quantitative data collected by project pharmacists to analyse the process of the integrating pharmacists is general practice. Framework analysis of the interview transcripts was used to align the results with the key process evaluation themes of implementation, mechanism of impact and context. Preliminary quantitative data was used to provide implementation feedback and to support the qualitative findings.

**Results:**

The interventional design included three phases, patient recruitment and selection, the pharmacist consultation and the communication and recording of recommendations. A number of barriers and facilitators affecting implementation were identified. Insight into the situational context of the intervention was gained from examining the differences between individual pharmacists and between practice sites.

**Conclusion:**

Conducting a process evaluation in the pilot phase of an integrated care project can allow adjustments to be made to the project procedures to improve the effectiveness and reproducibility of the intervention going forward.

## Introduction

Traditionally phamacists in Australia have practiced in the hospital or community pharmacy setting. The integration of pharmacists in general practice is an example of an inter-professional collaborative intervention that has previously been demonstrated to improve both health and economic outcomes [1,2]. This emerging area of professional practice provides a novel opportunity for pharmacists to demonstrate their cognitive pharmacotherapy skills and utilise team-based care. The proposed role of the general practice pharmacist includes not only providing direct medication management services to patients but may also include review of general practice prescribing and disease state management [3].

Previous studies [4,5,6] have identified factors that can affect the implementation of inter-professional interventions involving general practice pharmacists including the importance of pharmacists being co-located with the general practice team, pharmacists demonstrating positive characteristics including adaptability and proactivity and pharmacists ensuring that they avoid negatively viewed roles such as diagnosing and dispensing.

There have been limited studies describing the components of interventions used by pharmacists integrated in general practice [7,8]. These studies did not evaluate or compare different aspects of the intervention components and there is subsequently no established best practice model for the integration of pharmacists in general practice.

In response to this evidence, and also to a perceived need for patient centred collaborative care, a Primary Health Network (administrative health region) in Western Sydney NSW, WentWest, has commissioned a project involving the integration of five pharmacists across twelve general practice sites with the pilot phase of the project beginning in March 2016. The target population of the project was patients at risk of medication misadventure with a focus on patients with complex medication regimens and/or multiple co-morbidities.

Healthcare interventions involving inter-professional collaboration are complex interventions. This is due to the involvement of multiple professional groups, the fact that the interventions involve many interacting components and also because they pose numerous implementation challenges. A systematic review by Supper et al. [9] demonstrated that inter-professional collaborative interventions have previously been associated with improvements in patient care across multiple contexts and professions. In contrast, a further systematic review by Schepman et al. [10] examined the common characteristics and outcomes of inter-professional collaborative interventions in primary health care and found that not all interventions were associated with positive health or economic outcomes.

Traditionally the evaluation of complex interventions has relied on reviewing study outcomes. This approach may lead to overlooking important implementation elements that may have contributed to how, or why, an intervention was successful and the influence of situational context on the intervention. More recently the use of process evaluation is being increasingly recognised as a valid technique for analysing complex interventions [12,13]. Conducting a process evaluation in an intervention’s pilot phase may help to identify potential issues and allow the adjustment of the interventional design to avoid future intervention outcome failure.

According to the Medical Research Council guidance, a process evaluation combines qualitative and quantitative methods to provide valuable insights into complex interventions. This is achieved by investigating (1) the mechanisms of impact used to achieve the intervention outcomes (2) the circumstances affecting how an intervention was implemented, and (3) how the situational context of the intervention affected its implementation and potential reproducibility [11]. This analysis provides important insight on the feasibility, appropriateness and acceptability of the intervention. These insights can then be used to assist future implementation planning and to improve reliability and reproducibility of outcomes by considering situational context and its impact.

When analysing the implementation of healthcare interventions, the Tailored Implementation for Chronic Diseases (TICD) checklist provides a framework for classifying barriers and facilitators to implementation. This checklist includes seven domains of factors affecting implementation. These include guideline factors, individual health professional factors, patient factors, professional interactions, incentives and resources, capacity for organisational change and social, political and legal factors [14].

The aim of this study was to conduct a preliminary process evaluation to inform the adaptation of the integrated pharmacist intervention.

## Description of care practice

### Methods

A mixed methods study was conducted to evaluate the process of integrating pharmacists in the Australian general practice setting, using a combination of semi-structured interviews with pharmacists and general practitioners (i.e., qualitative data) and an *ad-hoc* dataset created for the delivery of the project (i.e., quantitative data).

Qualitative data was collected using one-on-one semi-structured interviews conducted either by telephone or face to face. All five pharmacists and a convenience sample of general practitioners selected by WentWest were approached by the WentWest head office and asked to participate in an interview with a member of the UTS research team between May and July 2016. Participants who consented to be interviewed were then contacted by the UTS researcher to arrange the interview. According to the Medical Research Council (UK) framework for conducting process evaluations [1] interview questions were designed to elicit information to:

1. describe the interventional model used and its application in practice.
2. inform about circumstances that may have affected the implementation of the intervention; and,
3. understand the situational context of the practice site.

Table 1 details the interview questions and links these to the MRC (UK) themes.

**Table 1:** Semi-structured interview questions.

*Questions to pharmacists* (MRC-UK themes) Please outline the process used to identify and book patients to see the clinical pharmacist at the surgeries you service. (Description of Interventional Model) Does the process differ between patients or surgeries? (If so, please describe how.) What is the procedure you use when conducting patient consultations? (Description of Interventional Model) Does this procedure vary for different medical conditions or different surgeries? (If so, please describe how.) (Situational Context)
How are the results of the consultation recorded? (Description of Interventional Model) Please outline the procedure used for communicating the results of the patient/pharmacist consultation to the general practitioner: (Description of Interventional Model) Does this procedure differ at different surgeries? (If so, please describe how.) (Situational Context)
What barriers have you experienced that reduced your effectiveness in integrating with the practice? (Circumstances affecting implementation) What facilitators have you observed that have assisted your integration into the practice? (Circumstances affecting implementation).
*Questions to general practitioners* What are your overall impressions of the clinical pharmacist project? (Circumstances affecting implementation, Situational context)What activities would you like the clinical pharmacists to perform during their time at the surgery? (Description of Interventional Model) What is the preferred method for the clinical pharmacist to communicate their recommendations to you? (Description of Interventional Model) What barriers have your observed that may reduce the effectiveness of the project? (Circumstances affecting implementation)What issues do you think may reduce the ability of the clinical pharmacist to improve patient outcomes? (Circumstances affecting implementation)What facilitators have you observed related to the project? (Circumstances affecting implementation) What can you suggest that may improve the effectiveness of the clinical pharmacist project? (Circumstances affecting implementation)

As part of their usual practice, the pharmacists participating in the study collected quantitative patient data using a data collection spreadsheet (in Microsoft Excel 2010©) that was developed to support the delivery of the intervention by the WentWest project team. This data was used to provide further insight into the three aspects encompassed by a process evaluation (Table 2).

**Table 2:** Quantitative data fields used to inform the process evaluation.

Variable*	Process Evaluation Theme(s)	Description

Number of current medicines (prescription and non- prescription)	Interventional Model	These variables were used to provide information on patient demographics to allow evaluation of the selection and recruitment process and to establish if the recruited patients reflected the project target population.
Number of current comorbidities	Interventional Model	
Age	Interventional Model	
Number of medication cessation recommendations	Interventional Model	These variables informed the researchers of the activities conducted during the patient consultation and provided insight into the impact of the intervention.
Number of addition of new medication recommendations	Interventional Model	
Number of recommendations for dose reduction	Interventional Model	
Number of suspected ADR identified	Interventional Model	
Number of suspected drug interactions detected	Interventional Model	
Number of recommendations for dose increase	Interventional Model	
Number of recommendations actioned by GP	Circumstances affecting implementation Situational Context	This variable provided insight into the effectiveness of the intervention and the level of collaboration between different practitioners. It was also used to demonstrate the differences in acceptance of the intervention at different sites to inform the situational context.
Number of recommendations by pharmacist	Circumstances affecting implementation Situational Context	This variable provided information on the ability of the pharmacist to implement the intervention. Differences in this variable were used to demonstrate the differences between pharmacist practitioners in conducting the intervention.
Practice ID	Situational Context	This variable allowed the researchers to consider the data from different practice sites to inform the situational context.
Pharmacist ID	Situational Context	This variable allowed the researchers to identify different pharmacist practitioners to inform the situational context.

* This table describes selected variables that were used to inform the process evaluation and is not a comprehensive list of the variables collected.

Research ethics approval was obtained from the Human Research Ethics Committee at the University of Technology Sydney (ETH16-0689).

Data analysis: Qualitative data was analysed using framework analysis. Data was coded according to the MRC-UK process evaluation key components of description of interventional model/mechanisms of impact, circumstances affecting implementation and situational context. This coding was reviewed by two researchers and a consensus on categorisation of the qualitative data was reached.

To describe processes relating to the interventional model the research team allocated the qualitative data to three different components reflecting the journey for patients with the integrated pharmacist service. These components allowed for modification and adjustment of data in the data analysis process. These categories were (i) patient selection and recruitment, (ii) the pharmacist consultation and (iii) communication and recording of the pharmacist recommendations.

To describe factors affecting implementation, elements that could hinder (i.e., barriers) or enable (i.e., facilitators) the implementation of the service were identified (Table 3). The identified elements were distributed into five relevant domains identified using the comprehensive, integrated checklist of determinants of practice (TICD) developed by Flottorp et al. [14] including:

- guideline factors (information relating to clinical practice guideline characteristics, intervention characteristics, and innovation characteristics),
- individual health professional factors (information relating to knowledge, attributes and behavior of health practitioners),
- patient factors (information relating to patient barriers, needs, experience, knowledge, skills, attitude and compliance),
- professional interactions (information relating to healthcare inter-professional barriers, network communications and culture, system characteristics and environmental and social factors including social influences and context), and
- incentives and resources (information relating to financial support, resources and incentives).

**Table 3:** Consultation data March–June 2016 (n = 299 consultations).

Demographics	Average patient age (years)	69.5 ± 12.1
	Average number of patient co-morbidities	7.1 ± 2.4
	Average number of medications per patient (prescription and non-prescription)	9.6 ± 4.0
Pharmacist recommendations	Total number	807
	Number recorded as accepted	329*
	Medication dose reduction	147
	Medication Cessation	173
	Medication dose increase	47
	New medication added	85
	Suspected adverse drug reaction	85
	Potential drug interaction	78
	Other recommendations	192
Other actions	Detection and resolution of discrepancies in patient record	349

* Pharmacist 2 did not record the number of recommendations accepted.

Two domains from the original checklist by Flottorp et al. [14] were excluded after data analysis due to a lack of relevant data for evaluation of the domains these were:

f) capacity for organisational change and
g) social, political and legal factors.

Finally data relating to contextual factors and processes shaping how the intervention works were allocated to situational context.

Quantitative data collected from the pharmacists was then entered into the Statistical Package for Social Sciences (SPSS) for Windows Version 24.0 (IBM, New York, USA.) for analysis of descriptive statistics [15]. Means and standard deviations were calculated to summarise quantitative variables where relevant.

## Results

Qualititative data was collected from four pharmacists as one pharmacist had left the project.

Five of twenty participating general practitioners agreed to be interviewed. These general practitioners came from four separate practice sites.

1. Adaptation of interventional model by project practices.

**Figure 1:**
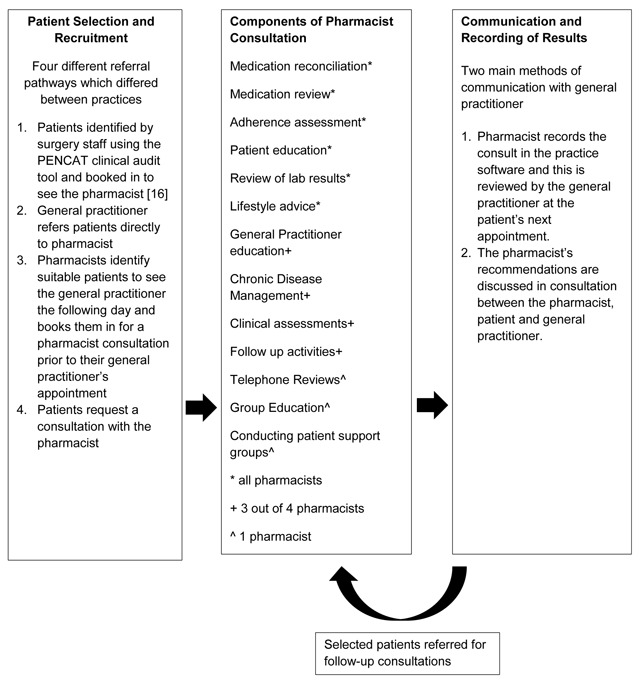
Intervention Model Design/Mechanisms of Impact.

Figure 1 depicts a summary of findings relating to the interventional model design adapted by each practice and includes three components:

- Patient Selection and recruitment.
  Patients were targeted for recruitment if they were taking more than five medications, were suspected of having an adverse reaction or medication adherence issues or required chronic disease management.
  Both pharmacists (n = 3) and general practitioners (n = 2) mentioned that the recruitment process worked best when the patients were identified by and booked in by the pharmacist. The reasons for this included that the pharmacist was motivated to recruit patients in contrast with other practice staff who saw this task as burdensome, that the pharmacist was most able to clearly articulate their role and identify potential benefits of the service for the patient and that the pharmacist was easily able to identify patients who would most benefit from the service.
- Pharmacist consultation.
  A total of 12 actions were undertaken as part of the patient consultation (Figure 1). All pharmacists performed medication review, medication reconciliation and a review of relevant lab results. Three of the four pharmacists conducted general practitioner education, chronic disease management, clinical assessment and organised follow up. Only one of the four pharmacists conducted group education, support groups and participated in patient telephone consultations.

This data provided evidence for the role of the pharmacist in conducting medication reconciliation and review and the identification of medication related problems as part of the patient consultation process.

iii) Communication and recording of recommendations.

All pharmacists had access to patient records and were able to document their recommendations in the patient record. Qualitative data collected in response to interview questions relating to communication of recommendations indicated that all pharmacists and general practitioners agreed that having a face to face interview was best. Ideally this would be a three way interview with the patient, pharmacist and general practitioner to agree on an action plan relating to the pharmacist’s recommendations. This was not always the practice implemented due to a lack of general practitioner availability.

Quantitative data from project pharmacists indicated that the process of recording consultation data was not consistent between pharmacists. Considerable variation in recording processes existed, for example, three of the five pharmacists included detailed descriptions of the recommendations made to general practitioners but two only recorded the number of recommendations made in each category without recording detail on agents involved.

Factors affecting implementation of the general practitioner-pharmacist intervention are outlined in Table 4.

**Table 4:** TICD implementation factors.

TICD Domain	Barrier	Facilitator

Guideline factors	Lack of guidelines, training and resources. Uncertain project timelines. Data collection spreadsheet design.	
Individual health professional factors	Individual general practitioners resistant to service. Individual pharmacist characteristics- lack of confidence and/or competence. Pharmacist perceived as a threat to the general practitioner’s professional territory.	General practitioners willing to collaborate. Positive professional relationship between pharmacist and general practitioner. Warm handover. Pharmacist proactive and clinically competent. Good communication between pharmacist and general practitioner.
Patient Factors	Patient resistance to service.	Improvement in patient outcomes due to ongoing follow up and review. Improved communication due to real time synchronous discussion. Doctor recommendation and introduction of the pharmacist reduced patient resistance
Professional Interactions	Lack of an established relationship between pharmacist and general practitioner and/or practice staff. Lack of general practitioner co-operation. Uncertainty regarding the role of the practice pharmacist. Resistance from community pharmacy. Pharmacist unable to establish rapport with other team members.	Team support.
Incentives and resources	Costs relating to the intervention. Lack of pharmacist remuneration and government funding for the service. Limited availability of the clinical pharmacist.	Allocation of sufficient funding. Increased pharmacist contact hours.

## Implementation Factors

### (a) Guideline factors

The data collection sheet designed by the project team was not comprehensive. For example, the data collection sheet did not include a field for the date of the consultation, the site of the consultation (relevant when they were visiting multiple sites) or the patient’s general practitioner. An additional limitation to the data collection method was that when a patient was seen more than once the data from all visits was recorded in the same line of the spreadsheet so it was not possible to determine which recommendations corresponded to which visit.

### (b) Individual health professional factors

Both pharmacists and general practitioners stated that the intervention works best when general practitioners are enthusiastic and willing to collaborate. General practitioners who actively recommended the pharmacist to the patient were seen as facilitators by several pharmacists and general practitioners. Several general practitioners mentioned that they thought the pharmacist should be both clinically competent and pro-active, and effective communication skills were identified as a facilitator.

At some sites despite participating in the project a lack of general practitioner co-operation from individual practitioners was seen as a barrier.

In addition uncertainty from practice staff, general practitioners and patients regarding the role of the pharmacist was felt to reduce the effectiveness of the intervention.

One general practitioner felt that they viewed the practice pharmacist as a threat to the general practitioner’s professional territory and was worried about the pharmacist’s activities eroding their role. GP1 “I think GPs assume that this is the start of a slippery slope where pharmacists will try to expand their role and encroach on the GPs territory.”

### (c) Patient Factors

The majority of pharmacists and one general practitioner stated that they had observed patient resistance to the service and that this was suggested to be a barrier to both recruitment of patients and the effectiveness of the intervention. In addition two pharmacists stated that they had difficulty recruiting patients to the service. Two general practitioners stated that ongoing contact with a pharmacist overcomes patient resistance to the service and improves outcomes. GP4 “Patients respond well to the accredited pharmacists manner.”

### (d) Professional Interactions

In the semi-structured interviews the lack of an established relationship between the individual pharmacist, general practitioner and the practice staff was raised as an initial barrier by all pharmacists and general practitioners. For example, Pharmacist 3 stated “I had no previous relationship with either of the GP’s and it took several weeks of consultations to establish my credibility.”

Several of the general practitioners communicated that they thought that their local community pharmacies would find the presence of the practice pharmacist threatening and were concerned about the impact of the intervention on their existing collaborative relationships with community pharmacy.

Both general practitioners and pharmacists indicated that support from the other members of the practice team improved the success of the intervention.

### (e) Incentives and resources

Costs relating to the intervention included the cost of the consultation room, software login and surgery utilities. One pharmacist mentioned that they had been employed for several weeks without receiving payment and that without reliable wages they were unlikely to continue working for the project.GP5 “The lack of grant money and ability to pay the pharmacist’s ongoing salary is a barrier to the service.”

The clinical pharmacists were often only present at practice sites for between four and eight hours per week. This limited availability was raised as a barrier to effective implementation of the service as the limited contact hours was seen to reduce the practice pharmacists’ potential impact. One pharmacist and two general practitioners mentioned that increasing the pharmacist contact hours increases collaboration and the effectiveness of the intervention.

## Situational context

There was no agreed protocol for the intervention across the project. As a result the method of patient selection and recruitment, the activities conducted by each practitioner and the way the results of the consultation were recorded and communicated varied between both practitioners and practice sites. One example is that four different methods were used for the selection and recruitment of patients.

The level of support for pharmacists provided by practices varied between sites. This included aspects of physical design (lack of a room, nameplate), the provision of support from other practice staff and provision of access to information and systems. This is illustrated in the case of practice software access where most sites provided the pharmacist with an individual login for practice software but at one site one pharmacist relied on the reception staff to log them into the practice software.

Data collection procedures varied between pharmacists and not all pharmacists accurately recorded all data fields. For example, one pharmacist did not complete the co-morbidity field, or the percentage of recommendations accepted. In addition not all pharmacists recorded detail about the recommendations made as a result of the consultation which reduced the information provided for analysis regarding the activities performed.

Table 5 outlines quantitative information differences in different practice sites and between different pharmacist practitioners, highlighting the variability in both the average number of recommendations made by different pharmacist practitioners and the differences in the percentage of recommendations accepted by general practitioners at various sites. The percentage of pharmacist recommendations accepted varied between practices as demonstrated by pharmacist four who had a 75% acceptance rate at one surgery and a 67% acceptance rate at the second surgery.

**Table 5:** Quantitative data informing the Situational context.

Pharmacist	Number of recommendations made per patient consultation (mean ± *standard deviation*)	Recommendations accepted by general practitioner n (%)

1	2.6 ± *2.0*	97 (94)
2	2.2 ± *1.8*	Not evaluable*
3	3.6 ± *1.4*	39 (91)
4	4.3 ± *1.4*	121 (72)
5	3.7 ± *1.6*	72 (92)

* Pharmacist 2 did not consistently record the number of recommendations accepted by the GP.

## Discussion

This study provides key information about how a newly implemented integrated healthcare intervention works in a real-world setting. By examining the mechanisms used to achieve the intervention outcomes, the circumstances affecting how the intervention was implemented, and how the situational context of the intervention affected its implementation, insights were gained to enable suggestions for improvement in processes for the project going forward. The lack of a standardised intervention procedure allowed comparison of the different approaches used between both pharmacists and different practice sites and this in turn increased the potential learnings available from the process evaluation.

In a systematic review of inter-professional collaborative interventions Supper et al. [9] found that a flexible model of care that was adapted for the setting and stakeholders received greater support from the team. The model of collaborative care used in the WentWest project appeared to support this premise as, although there were some common activities at each site, many of the processes and procedures conducted by the pharmacists varied depending on the requirements of each practice and the health care professionals and patients involved.

The professional relationship between the GP, health professional and practice staff are identified as a key implementation consideration in previous studies [4,5,6] and this was supported by our study. Establishing the professional credibility of the health professional (in this case the pharmacist) and clearly describing their role to all collaborators may help to proactively assist the development of a collaborative professional relationship. Funding and system level support is essential in allowing not only successful initial implementation of an intervention but also for the longer term exploration and maintenance stages. Supper et al. [9] identified the lack of remuneration, long-term funding and physical space as a significant barrier for pharmacists. These findings are supported by the results of this evaluation in which several participants identified both the lack of government funding and the difficulty of allocating consultation space for the pharmacists as significant barriers to the provision of the integrated pharmacist service. Long term sustainability of the intervention relies on sufficient ongoing funding and any implementation plan should include a comprehensive funding model.

Previous studies examining roles and guidelines for pharmacists integrated in primary care teams have identified the importance of establishing the role of the pharmacist in accordance with the needs and priorities of the general practice team and patients. In addition, the importance of ensuring that pharmacists are clinically competent, highly visible and proactive was identified as requirement for successful integration [16,17]. These findings were supported by the process evaluation results where project pharmacists found patient recruitment and communication of recommendations to general practitioners worked best where the pharmacist role and professional competencies were clearly understood.

Differences in situational context including individual practitioner pharmacist characteristics and differences in setting were both found to be important in predicting the success of a pharmacist’s integration and effectiveness by Jorgensen et al. [6] who examined the differences in the success of inter-professional collaboration between 24 pharmacists integrated into primary care teams in Canada. In the WentWest project several differences were identified in both the qualitative and quantitative data between individual practitioner pharmacists and between different practice sites. Sometimes these differences were due to different procedures adopted by different practitioners illustrated by the differences in data recording procedures and in other instances the cause of the variation was more difficult to detect. Ensuring that pharmacists receive training in essential project procedures such as data collection and recording prior to the commencement of the project should increase the level of consistency of results between practitioners. Other contextual differences such as the ability of a pharmacist to proactively communicate and overcome barriers to collaboration are perhaps more difficult to address but should still be taken into account when planning an intervention.

This study highlights the importance of clearly defining and communicating an intervention’s components to all collaborators. In addition, ensuring that all staff are trained and provided with sufficient guidelines, resources and system level support will improve the consistency and reproducibility of an intervention’s delivery. Establishing health practitioner competency and credibility and clearly defining individual practitioner roles will assist with improving the effectiveness of a collaborative intervention. In addition, effective inter-professional communication between all collaborators will improve the success of complex interventions involving multiple health practitioners.

This study was limited by the time and resources available to the research team. As a result the sample size of general practitioners interviewed was limited to five out of a potential 20 general practitioners and this may have meant that data saturation was not reached and there are further potential learnings that have not been presented in the data. However, the information gathered from the interviews was sufficient to allow for the identification of the main themes. It recommended that further process evaluation is conducted at later stages of the project to ensure that the learnings from the study are comprehensive and allow for further adjustment of the model and implementation plan where required.

This process evaluation has provided insight into the potential impact of pharmacists in general practice. Additional research is required, and currently underway examining the economic, humanistic and clinical outcomes resulting from the integration of general practice pharmacists.

## Conclusion

Conducting a process evaluation in the pilot phase of a complex intervention is particularly relevant as it enables the intervention model to be adapted to reduce the chance of future intervention failure. Addressing relevant implementation barriers and facilitators, evaluating intervention model design and considering situational context can aid the development of a robust, reproducible intervention that is potentially less likely to fail in the exploration and sustainability phase. The analysis of both qualitative and quantitative data collected in the first twelve weeks of the WentWest non-dispensing pharmacist project by the UTS research team has allowed the authors to provide advice and insight to the project team and has resulted in the standardisation of the interventional model.

The results from this process evaluation were communicated to WentWest via an internal report and the UTS team assisted in implementing the report recommendations by liaising with the WentWest project team and by conducting a training day for all participating project pharmacists. As a result of this study adjustments have been made to the ongoing project including changes to patient selection and recruitment procedures, Activities conducted during the patient consultation and communication and recording of pharmacist recommendations.
